# Integrative gene network and functional analyses identify a prognostically relevant key regulator of metastasis in Ewing sarcoma

**DOI:** 10.1186/s12943-021-01470-z

**Published:** 2022-01-03

**Authors:** Florencia Cidre-Aranaz, Jing Li, Tilman L. B. Hölting, Martin F. Orth, Roland Imle, Stefanie Kutschmann, Giulia Ammirati, Katharina Ceranski, Martha Julia Carreño-Gonzalez, Merve Kasan, Aruna Marchetto, Cornelius M. Funk, Felix Bestvater, Simone Bersini, Chiara Arrigoni, Matteo Moretti, Uwe Thiel, Daniel Baumhoer, Felix Sahm, Stefan M. Pfister, Wolfgang Hartmann, Uta Dirksen, Laura Romero-Pérez, Ana Banito, Shunya Ohmura, Julian Musa, Thomas Kirchner, Maximilian M. L. Knott, Thomas G. P. Grünewald

**Affiliations:** 1 Hopp-Children’s Cancer Center (KiTZ), Heidelberg, Germany; 2 Division of Translational Pediatric Sarcoma Research (B410), German Cancer Research Center (DKFZ), German Cancer Consortium (DKTK), Im Neuenheimer Feld 280, 69210 Heidelberg, Germany; 3 Max-Eder Research Group for Pediatric Sarcoma Biology, Institute of Pathology, Faculty of Medicine, LMU Munich, Munich, Germany; 4 Soft-Tissue Sarcoma Junior Research Group, German Cancer Research Center (DKFZ), German Cancer Consortium (DKTK), Heidelberg, Germany; 5 Faculty of Biosciences, Heidelberg University, Heidelberg, Germany; 6 Division of Pediatric Surgery, Department of General, Visceral and Transplantation Surgery, University Hospital Heidelberg, Heidelberg, Germany; 7 Regenerative Medicine Technologies Laboratory, Ente Ospedaliero Cantonale (EOC), Lugano, Switzerland; 8 Light Microscopy Facility (W210), German Cancer Research Center (DKFZ), German Cancer Consortium (DKTK), Heidelberg, Germany; 9 Biomedical Sciences Faculty, Università della Svizzera Italiana (USI), Lugano, Switzerland; 10 Technical University of Munich, School of Medicine, Department of Pediatrics and Children’s Cancer Research Center, Munich, Germany; 11 Bone Tumor Reference Center, Institute of Pathology of the University Hospital of Basel, Basel, Switzerland; 12 Clinical Cooperation Unit (CCU) Neuropathology, German Cancer Research Center (DKFZ), German Cancer Consortium (DKTK), Heidelberg, Germany; 13 Department of Neuropathology, Institute of Pathology, Heidelberg University Hospital, Heidelberg, Germany; 14 Division of Pediatric Neuro-Oncology, German Cancer Research Center (DKFZ), German Cancer Consortium (DKTK), Heidelberg, Germany; 15 Division of Translational Pathology, Gerhard-Domagk-Institute for Pathology, University Hospital Münster, Münster, Germany; 16 Pediatrics III, AYA Unit, West German Cancer Centre, University Hospital Essen, Essen, Germany; 17 German Cancer Consortium (DKTK), partner site Essen, Essen, Germany; 18 Department of General, Visceral and Transplantation Surgery, Heidelberg University Hospital, Heidelberg, Germany; 19 Institute of Pathology, Heidelberg University Hospital, Heidelberg, Germany

The identification of cancer stemness genes is crucial to understand the underlying biology of therapy resistance, relapse, and metastasis. In pediatric tumors – despite being largely composed of undifferentiated stem cell-like cells – mutations generally do not involve canonical stemness/metastasis-associated genes [1]. Ewing sarcoma (EwS), the second most common bone cancer in children and adolescents [2], is a highly aggressive malignancy associated with dismal outcome in metastatic disease [3], wherefore deciphering mechanisms of metastasis is imperative. EwS is characterized by a remarkably ‘silent’ genome with a single driver mutation generating an oncogenic fusion transcription factor (*EWSR1-ETS*) that promotes stem cell features [4, 5]. Thus, EwS constitutes an ideal model to study how perturbation of a transcriptional network by a dominant oncogene can mediate metastasis.

To identify highly relevant *EWSR1-ETS* target genes involved in the stemness/metastasis axis we implemented a systems biology approach to analyze published transcriptome profiles of 5 EwS cell lines with/without shRNA-mediated knockdown of *EWSR1-FLI1* or *-ERG* (< 20%) for 96 h [6] as described in Fig. 1a*.* This analysis yielded 348 differentially expressed genes (DEGs) being up- or downregulated (|log2 FC| ≥ 1.5) after knockdown of *EWSR1-FLI1* or *-ERG* across all cell lines (Supplementary Table 1). We next filtered these DEGs for genes annotated with the significantly enriched gene ontology term ‘Regulation of Cell Differentiation’ using PantherDB (*P* = 4.93 × 10^− 12^, false-discovery rate = 1.97 × 10^− 9^). The resulting 76 DEGs (Supplementary Table 2) were subjected to network analysis for pathway, physical, and genetic interactions using *Cytoscape* and *GeneMania* (Fig. 1b, c). To identify clinically relevant key hubs within this network we focused on transcription factors (*n* = 11) that were highly interconnected (> 10 interactions) with a significant (*P* < 0.05, Mantel-Haenszel test) association with overall survival in a cohort of 166 EwS patients with matched gene expression and clinical data [7]. After adjustment for multiple testing (Supplementary Table 3), transcription factor 7 like 1 (*TCF7L1*, alias *TCF3*) emerged as the most promising candidate for functional follow-up, whose low expression was associated with poor patients’ overall survival (nominal *P* = 0.0057; *P* = 0.0228 Bonferroni-adjusted) (Fig. 1d). Next, we explored TCF7L1 protein levels in association with clinical outcome in tissue micro-arrays (TMAs) comprising 114 EwS tumors and found that low TCF7L1 protein expression was significantly (*P* = 0.035) associated with worse overall survival (Fig. 1e), supporting that low TCF7L1 expression promotes an aggressive phenotype. Notably, TCF7L1 appeared to be downregulated by EWSR1-ETS since microarray data of each respective fusion oncogene knockdown induced a ~3-fold upregulation of TCF7L1 (Supplementary Table 4), which was confirmed in 5 EwS cell lines by qRT-PCR (average ~ 6-fold upregulation) (Supplementary Fig. 1b) and validated in A673 cells xenografts with/without conditional knockdown of EWSR1-FLI1 (A673/TR/shEF1 cells) at mRNA and protein levels (Supplementary Fig. 1c).

**Fig. 1:**
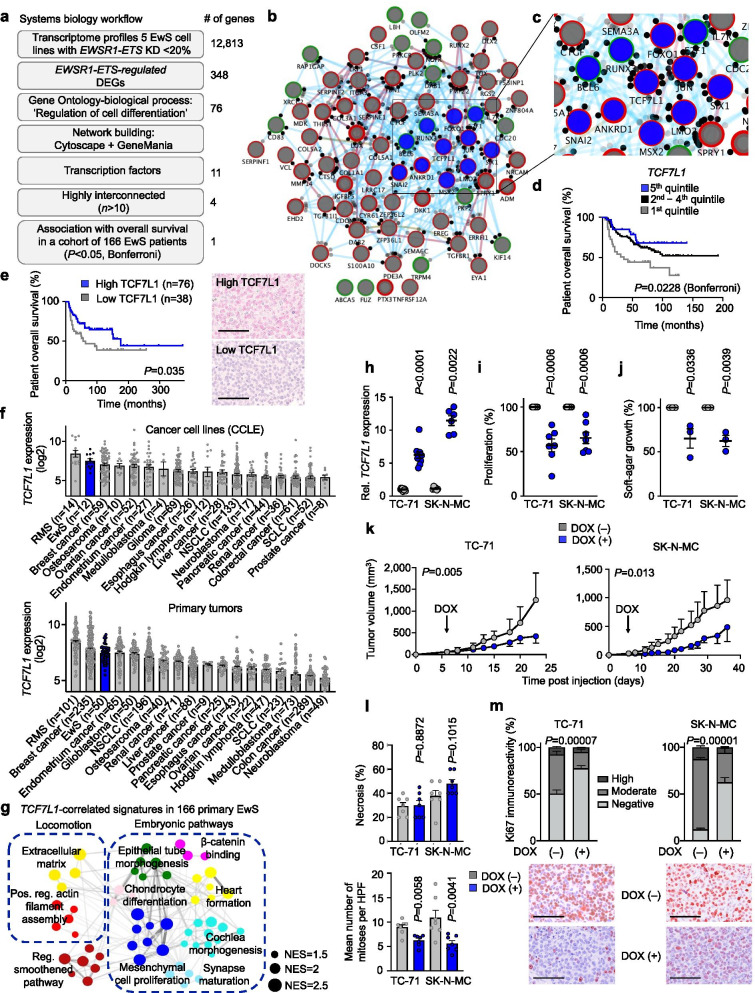
Systems biology identifies TCF7L1 as a prognostically relevant EWSR1-ETS-regulated network hub whose re-expression inhibits tumorigenesis in vitro and in vivo. **a)** Workflow depicting a systems biology approach to identify DEGs regulated by EWSR1-ETS, involved in regulation of cell differentiation, functioning as highly interconnected TFs, and associated with overall survival in a cohort of 166 EwS patients. Number of genes represent remaining candidates after each filtering step. **b)** Network of EWSR1-ETS-regulated genes involved in regulation of cell differentiation. Genes are depicted as nodes (circles). Blue nodes represent TFs, node outline color show up- (green) or down- (red) regulation by EWSR1-ETS. Node border width represent strength of regulation by EWSR1-ETS (the thicker the border the higher the fold-change). Black dots surrounding the nodes represent interconnections with other nodes within the network. Connecting lines show three types of interconnection: physical (red), pathway (blue), genetic (brown). Line width represents strength of interconnection. **c)** Close-up image of highly interconnected TFs located at the center of the network (blue nodes). **d)** Kaplan-Meier survival analysis of 166 primary EwS patients stratified by quintile *TCF7L1* expression. Mantel-Haenszel test, Bonferroni corrected. DEG: differentially expressed genes; KD: knockdown. **e)** Kaplan–Meier survival analysis of 114 EwS patients stratified by TCF7L1 protein expression intensity (low intensity≤1, high intensity> 1). *P*-value was determined by Gehan-Creslow-Wilcoxon test. Representative micrographs of TCF7L1 IHC are depicted. Scale bar = 50 μm. **f)** *TCF7L1* expression in cell lines or primary tumors from EwS and 17 additional tumor entities (Cancer Cell Line Encyclopedia, CCLE). Data are represented as bar plots where horizontal bars represent mean and SEM. The number of samples per group (*n*) is given in parentheses. RMS, rhabdomyosarcoma; NSCLC, non-small cell lung carcinoma; SCLC: small-cell lung carcinoma. **g)** Weighted Gene Correlation Network Analysis (WGCNA) of enriched gene-sets obtained by Pearson correlation analysis of genes whose expression is negatively correlated with *TCF7L1* expression in Affymetrix expression data of 166 EwS tumors. Network depicts signatures presenting NES > 1.5 and *P* < 0,05. NES, normalized enrichment score. **h)** Relative *TCF7L1* expression (qRT-PCR) of TC-71 and SK-N-MC cells containing a DOX-inducible re-expression construct for *TCF7L1*. Cells were grown either with or without DOX for 72 h. *n* ≥ 6 biologically independent experiments. Two-sided Mann-Whitney test. **i)** Viable cell count of TC-71 and SK-N-MC cells containing a DOX-inducible *TCF7L1* re-expression construct 72 h after DOX-treatment. Data are mean and SEM, *n* = 7 biologically independent experiments. Two-sided Mann-Whitney test. **j)** Relative area covered by colonies (%) grown in soft-agar of EwS cells containing a DOX-inducible re-expression construct for *TCF7L1*. Cells were grown either with/without DOX-treatment. *n* = 3 biologically independent experiments. Two-sided Mann-Whitney test. **k)** Growth of subcutaneous xenografts of indicated EwS cells containing a DOX-inducible *TCF7L1* re-expression construct (arrow = start of DOX-treatment). Data are represented as means (*n* = 7 animals/group). Two-sided Mann-Whitney test. **l)** Ex vivo analysis of relative necrotic area (top) and mitotic index (bottom) of xenografted TC-71 and SK-N-MC cell lines. Data are mean and SEM, *n* = 7 animals/group. **m)** Ex vivo analysis of Ki67 positivity of xenografted TC-71 and SK-N-MC cell lines. Horizontal bars represent means and whiskers SEM, *n* = 7 animals/group. *P*-values were determined via χ^2^ test testing all positives (high and moderate immunoreactivity) versus negatives. Histological images depict representative Ki67 micrographs. Scale bar = 50 μm.

Prior reports suggested that *TCF7L1* may have either oncogenic or tumor suppressor properties depending on the cellular context. Indeed, *TCF7L1* expression has been linked to promotion of cell proliferation, tumor growth and sphere formation in breast cancer [8], colorectal cancer [9, 10], acute lymphoblastic leukemia [11] and skin squamous cell carcinoma [12], while ectopic expression of *TCF7L1* inhibits self-renewal of liver cancer stem cells [13]. To explore its role in EwS, we first analyzed the *TCF7L1* expression pattern across 18 cancer entities using microarray data from the Cancer Cell Line Encyclopedia (CCLE) and an own study that compiled well-curated microarray data from the same cancer entities [14]. Surprisingly, *TCF7L1* was very highly, but variably, expressed in EwS cell lines and primary tumors (Fig. 1f).

Weighted Gene Correlation Network Analysis (WGCNA) based on enriched gene-sets in *TCF7L1-*correlated genes in 166 EwS tumors showed that EwS tumors with low *TCF7L1* expression were enriched in embryonic pathways (Fig. 1g). Although *TCF7L1* is generally highly expressed in EwS, these data suggested that suppression of its transcription by EWSR1-FLI1 is associated with embryonic processes that may be linked with poor patient outcome. Thus, we generated two EwS cell lines (SK-N-MC and TC-71, with lowest *TCF7L1* expression across the 5 EwS cell lines tested (Supplementary Fig. 1a) with a doxycycline- (DOX)-inducible re-expression of *TCF7L1* (Fig. 1h)*.* Consistent with the hypothesis that *TCF7L1* may be an EWSR1-ETS-repressed downstream transcription factor (with a potentially indirect regulation, possibly partially mediated by *TCF7L1* promoter hypermethylation as depicted in Supplementary Fig. 1d–g), transcriptome profiling of two EwS cell lines after either knockdown of EWSR1-ETS or re-expression of *TCF7L1* showed a highly significant (*P* = 2.57 × 10^− 165^ or *P* = 5.34 × 10^− 272^) overlap of concordant DEGs (Supplementary Fig. 1e). Congruently, conditional re-expression of *TCF7L1* significantly reduced cell proliferation (Fig. 1i), clonogenic growth in two-dimensional (2D) cultures (Supplementary Fig. 2b), anchorage-independent and three-dimensional (3D) growth (Fig. 1j), and local tumor growth in a pre-clinical subcutaneous xenotransplantation mouse model (Fig. 1k). The xenografts exhibiting specific re-expression of TCF7L1 (DOX(+) group) (Supplementary Fig. 2e,f) showed a reduced mitotic index (Fig. 1l), and a significant decrease of the proliferation marker Ki67 (Fig. 1m). Similar in vitro and in vivo experiments performed with both cell lines ‘empty’ controls exhibited no significant differences (Supplementary Fig. 2a, c-d, g-i).

Since stemness features, such as elevated clonogenic capacity and anchorage-independent growth, are essential for circulating tumor cells to colonize and develop into clinically apparent metastases in distant organs, we reasoned that *TCF7L1* may be linked to the metastatic process in EwS. In support of this hypothesis, transcriptome profiling, subsequent gene-set enrichment and WGCNA of *TCF7L1*-reexpressing SK-N-MC and TC-71 EwS cells uncovered that low *TCF7L1* levels led to overrepresentation of gene-sets involved in cellular migration (Fig. 2a). Accordingly, re-expression of *TCF7L1* resulted in significantly reduced transwell migration (Fig. 2b), invasion, and single-cell 3D migration in fibrin gel using advanced microfluidic chambers (Fig. 2c). Next, we employed an orthotopic spontaneous in vivo metastasis model by injecting *TCF7L1* re-expressing cells into the proximal tibiae of immunocompromised mice. Remarkably, while there was no significant difference in local tumor growth in the limited space of the tibial plateau (Supplementary Fig. 3a,b), we observed a strong inhibition of macrometastatic spread to liver, lungs, and kidneys upon re-expression of *TCF7L1*, and significantly reduced micrometastatic burden in the same organs (Fig. 2d,e, Supplementary Fig. 3c–e). In agreement with these in vivo findings, we observed that *TCF7L1* was significantly (*P* = 0.016) lower expressed in EwS metastases (*n* = 7) compared to primary tumors (*n* = 118) in situ as defined by RNA-sequencing of tumor tissue (Fig. 2f). Strikingly, this result became even more significant when focusing on gene expression data of 4 matched pairs of metastases and primaries (*P* = 0.0078) (Fig. 2g).

**Fig. 2:**
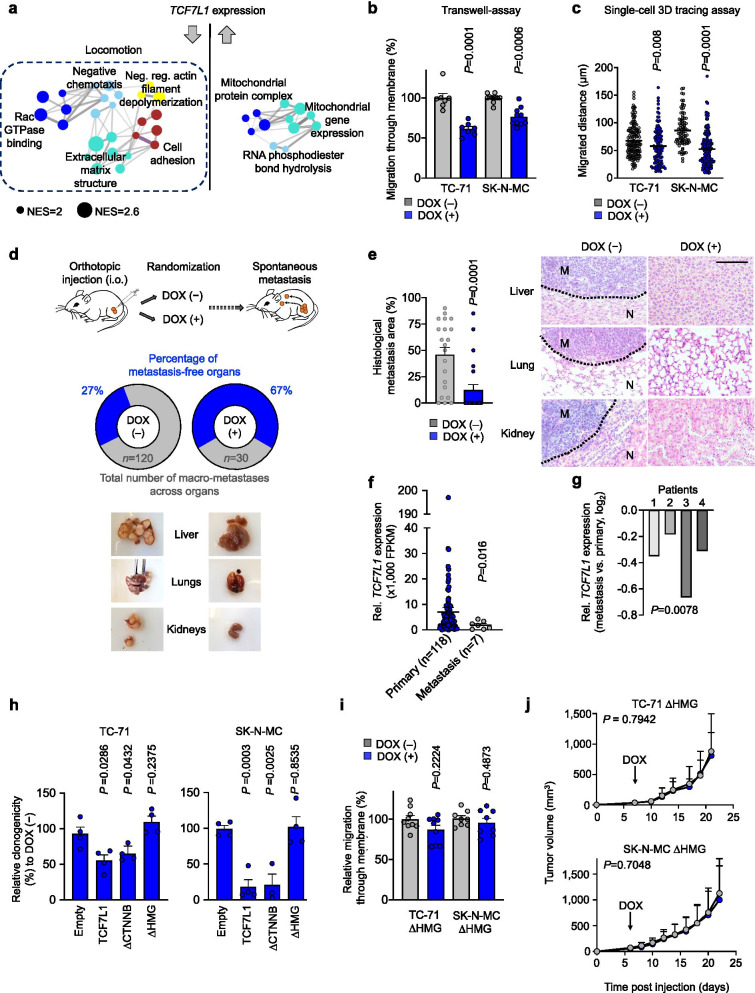
**| High TCF7L1 expression inhibits metastasis in EwS through its DNA binding domain. a)** Weighted Gene Correlation Network Analysis (WGCNA) depicting functional gene enrichment of down- or up-regulated genes in *TCF7L1* re-expressing EwS cells. Network depicts signatures presenting *P* < 0,05, NES > 2. NES, normalized enrichment score. Arrows depict direction of gene regulation. **b)** Relative percentage of migrated cells in 6 h. TC-71 and SK-N-MC EwS cells containing a DOX-inducible re-expression construct for *TCF7L1* where pre-treated with or without DOX for 72 h. *n* ≥ 7 biologically independent experiments. Two-sided Mann-Whitney test. **c)** Invasion and single cell migrated distance in 15 h. TC-71 and SK-N-MC EwS cells containing a DOX-inducible re-expression construct for *TCF7L1* where pre-treated with or without DOX for 72 h and added to a microfluidic chamber containing a fibrin compartment. *n* ≥ 3 biologically independent experiments. Two-sided unpaired t-test. **d)** Schematic representation of the experimental design: TC-71 or SK-N-MC EwS cell lines containing a DOX-inducible re-expression construct for *TCF7L1* were injected in the right tibia plateau. Animals were subsequently randomized and treated with or without DOX. At the end of the experiment, mice were evaluated ex vivo for presence of spontaneous metastases in inner organs. Pie charts depict percentage of metastasis-free organs (blue) in each condition, *n* represents total number of metastasis. Bottom pictures show representative images of metastatic organs in DOX(−) and DOX(+) conditions. *n* = 8 animals/group. **e)** Graph depicts relative area of histological metastatic spread of orthotopically injected SK-N-MC EwS cells containing a DOX-inducible re-expression construct for *TCF7L1*. *n* = 21 slides/group. Two-sided Mann Whitney test. Pictures show representative histological images of HE stainings from the evaluated organs. Scale bar is 50 μm. M, metastasis; N, normal tissue. **f)** Comparison of relative *TCF7L1* expression of EwS primary tumors versus metastasis (*n* = 125), normalized using *RPLP0* expression of the same samples. **g)** Analysis of relative *TCF7L1* expression (normalized to *RPLP0* expression of the same samples) in paired metastasis/primary samples from EwS patients 1–4. Independent one sample t-test. **h)** Relative colony number of colony-forming assays (CFAs) of TC-71 (left) and SK-N-MC (right) cells containing a DOX-inducible re-expression construct for *TCF7L1*, empty control, or one of the two deletion mutants for *TCF7L1* (deletion mutant for the β-catenin binding domain, ∆CTNNB; deletion mutant for the DNA binding domain, ∆HMG). Cells were grown either with or without DOX. *n* = 4 biologically independent experiments. **i)** Relative percentage of migrated cells in 6 h. TC-71 and SK-N-MC EwS cells containing a DOX-inducible re-expression construct for *TCF7L1* DNA binding domain (∆HMG) where pre-treated with or without DOX for 72 h. *n* ≥ 8 biologically independent experiments. **j)** Growth of EwS subcutaneous xenografts of TC-71 and SK-N-MC cells containing a DOX-inducible re-expression construct for *TCF7L1* deletion mutant ∆HMG (arrow indicates start of DOX treatment). Data are represented as means (*n* = 7 animals/group). Two-sided Mann-Whitney test

To further investigate the mechanism of action of *TCF7L1*, we generated two EwS cell lines with conditional re-expression of different *TCF7L1* deletion mutants for its major domains (ΔHMG: DNA binding domain; ΔCTNNB: β-catenin binding domain) and observed that only the ΔHMG mutants exhibited a normal clonogenic growth and migration capacity in vitro (Fig. 2h,i), and tumor growth in vivo (Fig. 2j). Moreover, further analysis of our WCGNA on *TCF7L1*-regulated signatures displayed in Fig. 2a highlighted several downregulated ‘driver target genes’ (aka leading-edge genes), including *ANXA1*, *LMO7*, *SLC9A9*, and *TMEM71* as potential key mediators of *TCF7L1* inhibition of the EwS migratory phenotype. These genes were selected for validation as they ranged among the top 10 downregulated genes by TCF7L1 and as their upregulation has been previously implicated in cancer progression [15–18]. As shown in Supplementary Fig. 3f, these genes are strongly downregulated upon re-expression of wildtype *TCF7L1* in both EwS cell lines tested, but remain unaltered in the DNA-binding-domain deletion mutant (ΔHMG), further suggesting that DNA-binding of *TCF7L1* is required to suppress the tumorigenic and migratory phenotype of EwS cells.

## Conclusions

Previous studies proposed a binary model of EWSR1-FLI1^-high^ promoting proliferation and EWSR1-FLI1^-low^ promoting metastasis [19] following a traditional epithelial-mesenchymal-transition concept. However, our findings support an integrative concept of the ‘migratory stem cell’ [20]: EwS cells may reside in a ‘metastable’ state where all EwS cells of a tumor may be equipped with both proliferative and migratory capacities [21]. Upon different intrinsic/extrinsic cues (of which EWSR1-FLI1 may not be the only important component) these cells could underlie a certain phenotypic accentuation.

In this context, we demonstrated that TCF7L1 is a critical mediator of metastasis in EwS, which may be utilized as a prognostic biomarker. Since we could detect a strong inverse correlation of TCF7L1 levels with patients’ overall survival in both the mRNA- as well as in the TMA-cohort, we believe that the most straightforward possibility to translate TCF7L1 into a routine clinical setting would be the IHC detection and semiquantitative evaluation of TCF7L1 in primary biopsies. This technique is readily available, inexpensive, and the conditions of IHC staining of TCF7L1 in EwS tumors have been established in the current study, which would offer the possibility of further evaluating the prognostic value of TCF7L1 in ongoing and prospective clinical trials. In addition, it would be highly interesting to explore whether TCF7L1 may serve as a prognostic biomarker in other (*EWSR1*-rearranged) mesenchymal neoplasms, which is subject to future studies.

In summary, our study exemplifies the power of systems biology to decipher gene regulatory networks and to identify key players in the metastatic process, which may be highly relevant for, and translatable to, other oligomutated (pediatric) cancers.

## Supplementary Information

**Additional file 1:**

**Additional file 2:**

**Additional file 3:**

## Data Availability

Original microarray data that support the findings of this study were deposited at the National Center for Biotechnology Information (NCBI) GEO and are accessible through the accession number GSE165929. Custom code is available from the corresponding author upon reasonable request.

## Abbreviations

CCLE: Cancer Cell Line Encyclopedia
CTNNB: Beta catenin
DOX: Doxycycline
DEG: Differentially expressed genes
EMT: Epithelial to mesenchymal transition
ETS: E26 transformation specific
EwS: Ewing sarcoma
EWSR1: Ewing sarcoma breakpoint region 1
ERG: ETS transcription factor ERG
FDR: False discovery rate
FET: FUS, EWSR1, TAF15
FC: Fold change
FFPE: Formalin-fixed and paraffin-embedded
FLI1: Friend leukemia virus integration 1
GO: Gene ontology
GSEA: Gene set enrichment analysis
HE: Hematoxylin Eosin
HMG: High mobility group
IHC: Immunohistochemistry
IRS: Immune reactive score
MTT: 3-[4,5-dimethylthiazol-2-yl]-2,5 diphenyl tetrazolium bromide
NES: Normalized enrichment score
NSG: NOD/SCID/gamma
TCF3: T-cell factor 3
TCF7L1: Transcription factor 7 like 1
TF: Transcription factor
TMA: Tissue microarray
ORF: Open reading frame
WGCNA: Weighted gene co-expression network analysis
Wnt: Wingless/Integrated

## References

[1] Lawrence MS, Stojanov P, Polak P, Kryukov GV, Cibulskis K, Sivachenko A (2013). Mutational heterogeneity in cancer and the search for new cancer-associated genes. Nature..

[2] Grünewald TGP, Cidre-Aranaz F, Surdez D, Tomazou EM, de Álava E, Kovar H, et al. Ewing sarcoma. Nat Rev Dis Primer. 2018 05;4(1):5. 10.1038/s41572-018-0003-x 29977059

[3] Thiel U, Wawer A, Wolf P, Badoglio M, Santucci A, Klingebiel T (2011). No improvement of survival with reduced- versus high-intensity conditioning for allogeneic stem cell transplants in Ewing tumor patients. Ann Oncol Off J Eur Soc Med Oncol.

[4] Tirode F, Laud-Duval K, Prieur A, Delorme B, Charbord P, Delattre O (2007). Mesenchymal stem cell features of Ewing tumors. Cancer Cell.

[5] Riggi N, Suvà M-L, De Vito C, Provero P, Stehle J-C, Baumer K (2010). EWS-FLI-1 modulates miRNA145 and SOX2 expression to initiate mesenchymal stem cell reprogramming toward Ewing sarcoma cancer stem cells. Genes Dev.

[6] Orth MF, Surdez D, Marchetto A, Grossetête S, Gerke JS, Zaidi S, et al. Systematic multi-omics cell line profiling uncovers principles of Ewing sarcoma fusion oncogene-mediated gene regulation [Internet]. 2021 Jun [cited 2021 Aug 16] p. 2021.06.08.447518. Available from: https://www.biorxiv.org/content/10.1101/2021.06.08.447518v1 10.1016/j.celrep.2022.111761 PMC10333306 36476851

[7] Musa J, Cidre-Aranaz F, Aynaud M-M, Orth MF, Knott MML, Mirabeau O, et al. Cooperation of cancer drivers with regulatory germline variants shapes clinical outcomes. Nat Commun. 2019 11;10(1):4128. 10.1038/s41467-019-12071-2 PMC6739408 31511524

[8] Slyper M, Shahar A, Bar-Ziv A, Granit RZ, Hamburger T, Maly B (2012). Control of breast cancer growth and initiation by the stem cell-associated transcription factor TCF3. Cancer Res.

[9] Murphy M, Chatterjee SS, Jain S, Katari M, DasGupta R. TCF7L1 Modulates Colorectal Cancer Growth by Inhibiting Expression of the Tumor-Suppressor Gene EPHB3. Sci Rep. 2016 23;6:28299. 10.1038/srep28299 PMC4917863 27333864

[10] Eshelman MA, Shah M, Raup-Konsavage WM, Rennoll SA, Yochum GS (2017). TCF7L1 recruits CtBP and HDAC1 to repress DICKKOPF4 gene expression in human colorectal cancer cells. Biochem Biophys Res Commun.

[11] Ma H, Mallampati S, Lu Y, Sun B, Wang E, Leng X (2014). The Sox4/Tcf7l1 axis promotes progression of BCR-ABL-positive acute lymphoblastic leukemia. Haematologica..

[12] Ku AT, Shaver TM, Rao AS, Howard JM, Rodriguez CN, Miao Q (2017). TCF7L1 promotes skin tumorigenesis independently of β-catenin through induction of LCN2. eLife..

[13] Shan J, Shen J, Wu M, Zhou H, Feng J, Yao C (2019). Tcf7l1 acts as a suppressor for the self-renewal of liver Cancer stem cells and is regulated by IGF/MEK/ERK signaling independent of β-catenin. Stem Cells Dayt Ohio.

[14] Baldauf MC, Orth MF, Dallmayer M, Marchetto A, Gerke JS, Rubio RA (2018). Robust diagnosis of Ewing sarcoma by immunohistochemical detection of super-enhancer-driven EWSR1-ETS targets. Oncotarget..

[15] de Graauw M, van Miltenburg MH, Schmidt MK, Pont C, Lalai R, Kartopawiro J (2010). Annexin A1 regulates TGF-beta signaling and promotes metastasis formation of basal-like breast cancer cells. Proc Natl Acad Sci U S A.

[16] Liu X, Yuan H, Zhou J, Wang Q, Qi X, Bernal C (2021). LMO7 as an unrecognized factor promoting pancreatic Cancer progression and metastasis. Front Cell Dev Biol.

[17] Ueda M, Iguchi T, Masuda T, Komatsu H, Nambara S, Sakimura S (2017). Up-regulation of SLC9A9 promotes Cancer progression and is involved in poor prognosis in colorectal Cancer. Anticancer Res.

[18] Wang K-Y, Huang R-Y, Tong X-Z, Zhang K-N, Liu Y-W, Zeng F (2019). Molecular and clinical characterization of TMEM71 expression at the transcriptional level in glioma. CNS Neurosci Ther.

[19] Franzetti G-A, Laud-Duval K, van der Ent W, Brisac A, Irondelle M, Aubert S (2017). Cell-to-cell heterogeneity of EWSR1-FLI1 activity determines proliferation/migration choices in Ewing sarcoma cells. Oncogene..

[20] Brabletz T, Jung A, Spaderna S, Hlubek F, Kirchner T (2005). Opinion: migrating cancer stem cells - an integrated concept of malignant tumour progression. Nat Rev Cancer.

[21] Sannino G, Marchetto A, Kirchner T, Grünewald TGP (2017). Epithelial-to-mesenchymal and mesenchymal-to-epithelial transition in mesenchymal tumors: a paradox in sarcomas?. Cancer Res.

