# Supplementary material for: Integrative gene network and functional analyses identify a prognostically relevant key regulator of metastasis in Ewing sarcoma
Source: Mol Cancer. 2022 Jan 3;21:1. doi: 10.1186/s12943-021-01470-z (PMC8722160; doi:10.1186/s12943-021-01470-z)
Supplement: Supplementary file 1 — Additional file 1: [file 12943_2021_1470_MOESM1_ESM.docx]

**METHODS**

**Provenience of cell lines and cell culture conditions**

Human EwS cell lines and other cell lines were provided by the following repositories and/or sources: SK-N-MC, TC-71, MHH-ES1, and RD-ES cells were provided by the German Collection of Microorganism and Cell Cultures (DSMZ). A673 and HEK293T were purchased from American Type Culture Collection (ATCC). TC106 cells were kindly provided by the Children’s Oncology Group (COG). A673/TR/shEF1 cells were kindly provided by J. Alonso (Madrid) (1). All cell lines were cultured in RPMI 1640 medium with stable glutamine (Biochrom, Germany) supplemented with 10% tetracycline-free fetal bovine serum (Sigma-Aldrich, Germany), 100 U/ml penicillin and 100 µg/ml streptomycin (Merck, Germany) at 37°C with 5% CO_2_ in a humidified atmosphere. Cell lines were routinely tested for mycoplasma contamination by nested PCR, and cell line identity was regularly verified by STR-profiling.

**RNA extraction, reverse transcription, and quantitative real-time polymerase chain reaction (qRT-PCR)**

Total RNA was isolated using the NucleoSpin RNA kit (Macherey-Nagel, Germany). 1 µg of total RNA was reverse-transcribed using High-Capacity cDNA Reverse Transcription Kit (Applied Biosystems, USA). qRT-PCR reactions were performed using SYBR green Mastermix (Applied Biosystems) mixed with diluted cDNA (1:10) and 0.5 µM forward and reverse primer (total reaction volume 15 µl) on a BioRad CFX Connect instrument and analyzed using BioRad CFX Manager 3.1 software. Gene expression values were calculated using the 2^−(ΔΔCt)^ method (2) relative to the housekeeping gene *RPLP0* as internal control. The thermal conditions for qRT-PCR were as follows: heat activation at 95 °C for 2 min, DNA denaturation at 95°C for 10 sec, and annealing and elongation at 60°C for 20 sec (50 cycles), final denaturation at 95°C for 30 sec. Oligonucleotides were purchased from MWG Eurofins Genomics (Germany) and are listed here:

*RPLP0* forward: 5’-GAAACTCTGCATTCTCGCTTC-3’

*RPLP0* reverse: 5’-GGTGTAATCCGTCTCCACAG-3’

*TCF7L1* forward*:* 5’-ATGAACGCCTCGATGTCC-3’

*TCF7L1* reverse*:* 5’-GGTTCCTGCTTGACGATGG-3’

*EWSR1-FLI1* forward: 5’-GCCAAGCTCCAAGTCAATATAGC-3’

*EWSR1-FLI1* reverse: 5’-GAGGCCAGAATTCATGTTATTGC-3’

*ANXA1* forward: 5’-AAGCAGGAGAAAGGAGAAAGG-3’

*ANXA1* reverse: 5’-AACTCCAGGTCCAGAACTTTG-3’

*TMEM71* forward: 5’-CATTGTTTCTCAGCCTTGCC-3’

*TMEM71* reverse: 5’-GATTCCAAGTAGACTGCAAGTTG-3’

*LMO7* forward: 5’-TGTTGCCTGTGAGTGTGAC-3’

*LMO7* reverse: 5’-ACAGTGCTTTCGTATGGAGG-3’

*SLC9A9* forward: 5’-ATGGTGTGGTCCGATTTCC-3’

*SLC9A9* reverse: 5’-TGGCTAGTTCATCCTGGTTTAC-3’

***TCF7L1* overexpression experiments**

# To re-express *TCF7L1* at physiological levels, we assessed the baseline expression levels of *TCF7L1* in 18 EwS cell lines for which whole-transcriptome data from Affymetrix Clariom D arrays was available (triplicates per cell line). As suitable models, we chose SK-N-MC and TC-71 cells as they exhibited the lowest baseline *TCF7L1* expression among these cell lines and proceeded with cloning as described in (3). Briefly, full-length cDNA of *TCF7L1* (NM_031283) was PCR-amplified from a commercial plasmid (Origene, SC126274) and an HA-tag was added by using AgeI- and NotI-restriction site containing primers (forward: 5’-ATTAACCGGTGCCACCATGCCCCAGCTCG-3’; reverse: 5’-TAATGCGGCCGCTTAAGCGTAATCTGGAACATCGTAGTGGGCAGACTTGGTGACC-3’) and a final Tm of 65°C (Phusion Polymerase, ThermoFisher), before cloning it into the multiple cloning site of a modified pTP vector (4). *TCF7L1* non-functional mutants were cloned from the same *TCF7L1* (NM_031283) ORF cDNA clone using touchdown-PCR. The HA-tagged CTNNB1-binding site truncated mutant was generated by using a different forward primer (5’-ATTAACCGGTGCCACCATGAACCAGAGCAGCAGCT-3’) and a final Tm of 63°C. The HMG-Box binding site was deleted by fusing two PCR products (Product 1, final Tm 57°C): forward: 5’-ATTAACCGGTGCCACCATGCCCCAGCTCG-3’; reverse: 5’-CTTACCATAGTTGTCGGGCTTCTTTTCCTCCT-3’; Product 2 (final Tm 64°C): forward: 5’-GAGGAAAAGAAGCCCGACAACTATGGTAAGAAAAAGAAGAGGA-3’; reverse: 5’-TAATGCGGCCGCTTAAGCGTAATCTGGAACATCGTAGTGGGCAGACTTGGTGACC-3’) using an overhang-extension PCR protocol (overhang-extension PCR (final Tm 65°C): forward: 5’-ATTAACCGGTGCCACCATGCCCCAGCTCG-3’; reverse: 5’-TAATGCGGCCGCTTAAGCGTAATCTGGAACATCGTAGTGGGCAGACTTGGTGACC-3’). The generated inserts were double restriction-digested with AgeI and NotI (NEB) and ligated into the pTP backbone (4) using T4 ligase (NEB). Positive clones were identified by colony PCR and cultured in 100 ml of LB Broth containing 100 µg/ml Ampicillin. Plasmids were extracted and purified using a Midi-Prep Kit (Macherey-Nagel). The correct insertion of full-length *TCF7L1* cDNA, empty control or *TCF7L1*-deficient mutants was verified by Sanger sequencing (sequencing primers: forward 5’-ACGTATGTCGAGGTAGGCGT-3’; reverse 5’-TTCGTCTGACGTGGCAGC-3’). Lentiviral particles were generated in HEK293T cells and used for transduction of SK-N-MC and TC-71 EwS cells using polybrene (8 µg/ml). Transduced cells were selected with 0.5 µg/ml puromycin. Re-expression of *TCF7L1* in SK-N-MC and TC-71 cells was achieved by addition of 1 µg/ml DOX to the culture medium. Cells were single-cell cloned and specific clones were selected and tested for re-expression of *TCF7L1*. Only the clones exhibiting re-expression levels similar to the expression FCs after *EWSR1-FLI1* silencing (see above) were selected for functional assays. Cells transfected with the empty vector were used as additional controls.

**Analysis of chromosomal gains, CNV and promoter methylation of *TCF7L1***

TCF7L1 mRNA expression data from EwS patient tumor samples was analyzed in association with CNV data of chr2/*TCF7L1* locus (2p11.2), and methylation status of *TCF7L1* promoter. These data were respectively derived from RNA sequencing (TruSeq RNA Exome (Illumina)), and methylation/CNV analysis (Infinium HumanMethylation450K BeadChip (EPIC array; Illumina)) from 115 EwS patient FFPE-samples. Since copy number gains or losses of the whole chromosome are calculated in average, we used the scores provided by the Integrative Genomics Viewer (IGV) for the region chr2:85,303,403-85,596,544. Estimation of the methylation status of the *TCF7L1* promoter was assessed by considering the CpG island located at locus chr2:85,359,957-85,362,593. Correlation analyses were performed with GraphPad Prism.

**Transcriptome analyses**

To assess the potential effect of *TCF7L1* on gene expression in EwS cells, microarray analysis was performed. To this end, 1.2×10^4^ cells per well were seeded in 6-well plates and treated with 1μg/μl DOX for 72h (DOX-refreshment after 48h). Thereafter, total RNA was extracted with the ReliaPrep miRNA Cell and Tissue Miniprep System (Promega) and RNA quality was assessed with a Bioanalyzer. All samples had an RNA integrity number (RIN)>9 and were hybridized to Human Affymetrix Clariom D microarrays. Gene expression data were quantile normalized with Transcriptome Analysis Console (v4.0; Thermo Fisher Scientific) using the SST-RMA algorithm as previously described (5). Annotation of the data was performed using the Affymetrix library for Clariom D Array (version 2, *Homo sapiens*) on gene level. DEGs with consistent and significant FCs across cell lines were identified as follows: Normalized gene expression signals were log2 transformed. To avoid false discovery artifacts due to the detection of only minimally expressed genes, we excluded all genes with a lower expression value than that observed for *ERG* of the respective cell lines (log2 expression signal of 7.08 for SK-N-MC and 6.57 for TC-71), which is known to be virtually not expressed in EWSR1-FLI1 positive EwS cell lines (6). The FCs of the control samples (empty vector) and both *TCF7L1* re-expressing EwS cell lines were calculated for each cell line separately. Then the FCs in the *TCF7L1* re-expressing samples were normalized to that of the empty control cells. Then both FCs were averaged to obtain the mean FC per gene across cell lines. DEGs were determined as having a log2 FC >0.5 or <–0.5, respectively.

**Gene-set enrichment analysis (GSEA)**

To identify enriched gene-sets, genes were ranked by their expression FC between the groups DOX (−) and DOX (+). GSEA was performed using the FGSEA R package (v 3.6.3) based on Gene Ontology (GO) biological processes terms from MSigDB (c5.all.v7.0.symbols.gmt) (7). GO terms were filtered for statistical significance (adjusted *P*<0.05) and a normalized enrichment score |(NES)|>2 (10,000 permutations). In order to construct a network, the Weighted Gene Correlation Network Analysis R package (WGCNA R) (8) was used. Briefly, a binary matrix of GO-terms × genes (where 1 indicates the gene is present in the GO term and 0 indicates it is not) was created. Then, the Jaccard’s distance for all possible pairs was computed to create a symmetric GO adjacent matrix. Clusters of similar GO terms were identified using dynamicTreeCut algorithm, and the top 20% highest edges were selected for visualization. The highest scoring node in each cluster was determined as the cluster label (rName). The obtained network and nodes files were fed into Cytoscape (v 3.8.0) for network design and visualization as previously described (9).

**Correlation analysis**

To identify gene-sets correlated with *TCF7L1* expression in primary tumors, we created a pre-ranked list of genes ordered by Pearson´s correlation coefficient with *TCF7L1* based on transcriptomic data of 166 of EwS (10) and GSEA was performed as described above.

**Proliferation assays**

For proliferation assays, 5–8×10^5^ EwS cells per well (depending on the cell line) were seeded in triplicates per group in 6-well plates and treated with 1 µg/ml DOX for 72h. Thereafter, cells including their supernatant were harvested and counted using standardized hemocytometers (C-Chip, Biochrom) and the Trypan-Blue (Sigma-Aldrich) exclusion method as described in (11).

**Clonogenic growth assays**

For clonogenic growth assays, *TCF7L1* re-expressing EwS cells and respective controls were seeded in triplicates at low density (2×10^3^ cells) per well in 12-well plates and grown for 9–11 days (depending on the cell line) with/without DOX-treatment (renewal of DOX or vehicle every 48h). Thereafter, colonies were stained with crystal violet (Sigma-Aldrich) and colony numbers and areas were measured with the ImageJ Plugin *Colony area*. The clonogenicity index was calculated by multiplying the counted colonies with the corresponding colony area.

**Sphere formation assays in soft agar**

For the analysis of anchorage-independent growth, *TCF7L1* re-expressing EwS cells and respective controls were pre-treated with/without DOX for 48h before seeding. A base of 2 ml of agar 1:1 with 2× DMEM medium was poured in wells of 6-well plates and left for 30–60 min to solidify. Then, 5×10^3^ cells per well were seeded in 500 µl in triplicates per condition (–/+DOX). Cells were kept in culture for 9–11 days and new medium (–/+DOX) was added on top of each well every 48h. Spheres were stained with 50 µl of a 5 mg/ml MTT solution that was added dropwise to each well and incubated for 1h. Pictures of the stained spheres were taken and their area was analyzed with ImageJ.

**Transwell assays**

For the analysis of migration through a porous membrane we proceeded as described in (12). Briefly, *TCF7L1* re-expressing EwS cells, their mutants, and respective controls were pre-treated with/without DOX for 72h before seeding and were starved by decreasing FCS concentration in the growth medium to 0.5% during the last 24h. The following morning, fresh medium with 10% FCS (chemoattractant) was plated in the lower compartment of a 24-well plate. Transwell attachments were placed on top and 1×10^5^ cells were plated on them with medium containing 0.5% FCS with/without DOX. Cells were allowed to migrate for 6h. After that, transwell inserts were washed with PBS and the top membrane was cleaned of remaining unmigrated cells with a cotton swab. Migrated cells were fixed with a 4% formaldehyde solution and stained with crystal violet. Transwells were washed with water to eliminate excess crystal violet and migrated cells were de-stained with a solution containing 2% acetic acid in PBS during 10 min on a shaker. The obtained solution was measured by absorbance at 570 nm in a plate reader.

**Microfluidic invasion and single cell 3D migration assay**

TC-71 and SK-N-MC *TCF7L1* re-expressing EwS cells were pre-treated for 48h with DOX (1µg/ml). Then, 1.5×10^6^ cells/ml were embedded in a 2.5 mg/ml fibrin matrix and introduced in the central channel of a microfluidic device as described (13). Following fibrin polymerization (20 min inside a humidity chamber), culture medium was added to the side channels of the microfluidic device. The next day, medium was substituted with CO_2_ independent culture medium (Thermo Fisher) and cells were imaged with an automated wide-field epifluorescence microscope (Lionheart FX, BioTek Instruments, Inc., Winooski, VT, USA). Images of at least 5 regions of interest for each microfluidic device per condition (*n*=3) were captured every 30 min for 15h. Temporal stacks were reconstructed using ImageJ and single cell 3D movement inside the fibrin matrix was quantified employing the image processing software Imaris (v.9.1, Bitplane AG, Zurich, Switzerland).

***In vivo* experiments in mice**

3×10^6^ SK-N-MC or TC-71 EwS cells harboring a re-expression construct for either *TCF7L1*, the *TCF7L1* deletion mutant (*TCF7L1*-HMGmut) or empty controls were injected in a 1:1 mix of cells suspended in PBS with Geltrex Basement Membrane Mix (ThermoFisher) in the right flank of 10–12 weeks old NOD/scid/gamma (NSG) mice as described in (14). Tumor diameters were measured every second day with a caliper and tumor volume was calculated by the formula L×l^2^/2, where L is the length and l the width. When the tumors reached an average volume of 80 mm^3^, mice were randomized in two groups of which one was henceforth treated with 2 mg/ml DOX (Beladox, Bela-pharm, Germany) dissolved in drinking water containing 5% sucrose (Sigma-Aldrich) to induce an *in vivo* re-expression (DOX (+)), whereas the other group only received 5% sucrose (control, DOX (−)). Once tumors of control groups reached an average volume of 1,500 mm^3^, all mice of the experiment were sacrificed by cervical dislocation. Other humane endpoints were determined as follows: Ulcerated tumors, loss of 20% body weight, constant curved or crouched body posture, bloody diarrhea or rectal prolapse, abnormal breathing, severe dehydration, visible abdominal distention, obese Body Condition Scores (BCS), apathy, and self-isolation. For analysis of EwS growth in bone, EwS cells were orthotopically injected into the proximal tibial plateau of NSG mice. To this end, one day before injection, mice were pre-treated with 800 mg/kg mouse weight/day Metamizole in drinking water as analgesia. On the day of injection, mice were anesthetized with inhaled isoflurane (1.5-2.5% in volume) and their eyes were protected with Bepanthen eye cream. After disinfection of the injection site, 2×10^5^ cells/20µl were directly injected with a fine 28 G needle (Hamilton, USA) into the right proximal tibia. For durable pain prophylaxis until the first day after intraosseous injection, mice were subsequently treated with Metamizole in drinking water (800 mg/kg mouse weight/day). At the first day after injection of tumor cells, mice were randomized in two groups of which one received henceforth 2 mg/ml DOX (BelaDox, Bela-pharm) dissolved in drinking water containing 5% sucrose (Sigma-Aldrich) to induce *TCF7L1* re-expression (DOX (+)), whereas the other group only received 5% sucrose (control, DOX (−)). All tumor-bearing mice were sacrificed by cervical dislocation at the predefined experimental endpoint, when the mice reached a humane endpoint as listed above or exhibited signs of limping at the injected leg (event) (43–52 days). After extraction of the tumors, a small fraction of each tumor was snap frozen in liquid nitrogen to preserve the RNA isolation, while the remaining tumor tissue was fixed in 4%-formalin and embedded in paraffin for immunohistology. In the case of the orthotopic model, all inner organs were harvested, weighed, photographed, 4%-formalin-fixed and embedded in paraffin for (immuno)histology. For analysis of the extent of metastatic spread, HE-stained histological slides were evaluated for presence of EwS cells and the area of metastasis versus total area was calculated.

**Immunohistochemistry (IHC) and immunoreactivity scoring (IRS)**

For IHC, 4-μm sections were cut and antigen retrieval was carried out by heat treatment with Target Retrieval Solution (S1699, Agilent Technologies) for Ki67, or Tris buffer pH 9.0 for TCF7L1 staining, respectively. For Ki67, the slides were stained with monoclonal anti-Ki67 raised in rabbit (1:200, 275R-15, Cell Marque/Sigma-Aldrich) for 60 min at RT, followed by a monoclonal secondary horseradish peroxidase (HRP)-coupled horse-anti-rabbit antibody (ImmPRESS Reagent Kit, MP-7401, Vector Laboratories). AEC-Plus (K3469, Agilent Technologies) was used as chromogen. For TCF7L1, the slides were stained with polyclonal anti-TCF7L1 antibody raised in rabbit (1:100 for complete slides, 1:25 for TMAs; 28835 clone D15G11 lot 5, Cell Signaling). For TMA analysis, DAKO REAL Detection System, Alkaline Phosphatase/RED Rabbit/Mouse was used (K5005, Dako). For xenograft slides, Biotin-SP-conjugated AffiniPure Goat Anti-Rabbit IgG (H+L) (111-065-144, Jackson Immunoresearch) and Alkaline Phosphatase Streptavidin (SA-5100, Vector) were used. The chromogen used was that included in the DAKO REAL Detection System (K 5005, Dako). Samples were counterstained with hematoxylin (H-3401, Vector Laboratories; or T865.3, Mayer, Roth). FFPE xenografts of the EwS cell lines were stained with HE for mitosis counting and metastatic spread assessment. Slides were scanned on a Nanozoomer-SQ Digital Slide Scanner (Hamamatsu Photonics K.K.) and visualized using NDP.view2 image viewing software (Hamamatsu Photonics K.K.). Mitoses were quantified by a blinded observer per high-power field (40×). Final scores/quantifications were determined by examination of 5–10 high-power fields of at least one section per sample. Evaluation of immunoreactivity of TCF7L1 was carried out in analogy to scoring of hormone receptors with a modified Immune Reactive Score (IRS) ranging from 0–12 that was adapted to EwS tumors (15,16). Briefly, the intensity of marker immunoreactivity was determined (grade 0=none, grade 1=low, grade 2=moderate and grade 3=strong). Slides were scored by *n*≥2 independent observers.

**Survival analysis**

Kaplan-Meier survival analyses were carried out in 166 EwS patients whose molecularly confirmed and retrospectively collected primary tumors were profiled at the mRNA level by gene expression microarrays in previous studies (17–20). To that end, microarray data generated on Affymetrix HG-U133Plus2.0, Affymetrix HuEx-1.0-st or Amersham/GE Healthcare CodeLink microarrays of the 166 EwS tumors (Gene Expression Omnibus (GEO) accession codes: [GSE63157](https://www.ncbi.nlm.nih.gov/geo/query/acc.cgi?acc=GSE63157) (17), [GSE12102](https://www.ncbi.nlm.nih.gov/geo/query/acc.cgi?acc=GSE12102) (18), [GSE17618](https://www.ncbi.nlm.nih.gov/geo/query/acc.cgi?acc=GSE17618) (19), [GSE34620](https://www.ncbi.nlm.nih.gov/geo/query/acc.cgi?acc=GSE34620) (20) provided with clinical annotations were normalized separately as previously described (15). Only genes that were represented on all microarray platforms were kept for further analysis. Batch effects were removed using the ComBat algorithm (21). Data processing was done in R.

**Comparative gene expression analysis from metastases and primary tumors**

Gene expression data from metastases and primary EwS tumor tissues was derived from two independent sources: I) selected samples from published Affymetrix Human Gene ST1.0 microarray data ([GSE45544](https://www.ncbi.nlm.nih.gov/geo/query/acc.cgi) and [GSE73166](https://www.ncbi.nlm.nih.gov/geo/query/acc.cgi?acc=GSE73166)) that were preprocessed using Robust Multiarray Average (22) and custom brainarray CDF (version 25) (23); II) RNA-sequencing data from archival tumor tissues of the Ewing 2008 study. Briefly, EwS FFPE tissue samples were evaluated to select tumor enriched areas (>70% of tumor cells) for nucleic acids isolation. RNA was extracted with Formapure Total kit (Beckman Coulter), following the recommended instructions. Quality and final quantification of RNA samples was evaluated on an Agilent 2100 Bioanalyzer (Agilent Technologies). For RNA sequencing, libraries were prepared according to TruSeq RNA Exome (Illumina) protocol and sequenced in a NextSeq 500 equipment (Illumina) as previously described (24). For each case, expression values of *TCF7L1* were normalized with each sample´s *RPLP0* expression value and an expression FC of metastasis/primary from the same patient was calculated.

**Analysis of published ChIP-seq data**

Publicly available and pre-processed ChIP-seq data from A673, TC-71, TC106, MHH-ES1 and SK-N-MC cells corresponding to EWSR1-FLI1 and H3K4me3 was retrieved, analyzed and displayed using R2 genomics analysis and visualization platform (25) for the TCF7L1 locus.

**Statistical analysis and software**

Statistical data analysis was performed using PRISM 9 (GraphPad Software Inc., Ca, USA) on the raw data. If not specified otherwise in the figure legends, comparison of two groups in functional *in vitro* experiments was carried out using a two-sided Mann-Whitney test. If not specified otherwise in the figure legends, data are presented as dot plots with horizontal bars representing means and whiskers representing the standard error of the mean (SEM). Sample size for all *in vitro* experiments were chosen empirically. For *in vivo* experiments, the sample size was predetermined using power calculations with *β*=0.8 and *α*<0.05 based on preliminary data and in compliance with the 3R principles (replacement, reduction, refinement). In Kaplan-Meier survival analyses, curves were calculated from all individual survival times of mice, respectively. Statistical differences between the groups were assessed by a Mantel-Haenszel test.

**REFERENCES**

1. Carrillo J, García-Aragoncillo E, Azorín D, Agra N, Sastre A, González-Mediero I, et al. Cholecystokinin down-regulation by RNA interference impairs Ewing tumor growth. Clin Cancer Res Off J Am Assoc Cancer Res. 2007 Apr 15;13(8):2429–40.

2. Livak KJ, Schmittgen TD. Analysis of relative gene expression data using real-time quantitative PCR and the 2(-Delta Delta C(T)) Method. Methods San Diego Calif. 2001 Dec;25(4):402–8.

3. Knott MML, Cidre-Aranaz F. Ewing Sarcoma-Specific (Re)expression Models. Methods Mol Biol Clifton NJ. 2021;2226:119–38.

4. Bauernfeind F, Rieger A, Schildberg FA, Knolle PA, Schmid-Burgk JL, Hornung V. NLRP3 inflammasome activity is negatively controlled by miR-223. J Immunol Baltim Md 1950. 2012 Oct 15;189(8):4175–81.

5. Marchetto A, Ohmura S, Orth MF, Knott MML, Colombo MV, Arrigoni C, et al. Oncogenic hijacking of a developmental transcription factor evokes vulnerability toward oxidative stress in Ewing sarcoma. Nat Commun. 2020 15;11(1):2423.

6. Crompton BD, Stewart C, Taylor-Weiner A, Alexe G, Kurek KC, Calicchio ML, et al. The genomic landscape of pediatric Ewing sarcoma. Cancer Discov. 2014 Nov;4(11):1326–41.

7. Subramanian A, Tamayo P, Mootha VK, Mukherjee S, Ebert BL, Gillette MA, et al. Gene set enrichment analysis: a knowledge-based approach for interpreting genome-wide expression profiles. Proc Natl Acad Sci U S A. 2005 Oct 25;102(43):15545–50.

8. Langfelder P, Horvath S. WGCNA: an R package for weighted correlation network analysis. BMC Bioinformatics. 2008 Dec 29;9:559.

9. Waszak SM, Robinson GW, Gudenas BL, Smith KS, Forget A, Kojic M, et al. Germline Elongator mutations in Sonic Hedgehog medulloblastoma. Nature. 2020 Apr;580(7803):396–401.

10. Musa J, Cidre-Aranaz F, Aynaud M-M, Orth MF, Knott MML, Mirabeau O, et al. Cooperation of cancer drivers with regulatory germline variants shapes clinical outcomes. Nat Commun. 2019 11;10(1):4128.

11. Funk CM, Musa J. Proliferation Assessment by Trypan Blue Exclusion in Ewing Sarcoma. Methods Mol Biol Clifton NJ. 2021;2226:151–8.

12. Cidre-Aranaz F. Analysis of Migration and Invasion in Ewing Sarcoma. Methods Mol Biol Clifton NJ. 2021;2226:167–79.

13. Bersini S, Lytle NK, Schulte R, Huang L, Wahl GM, Hetzer MW. Nup93 regulates breast tumor growth by modulating cell proliferation and actin cytoskeleton remodeling. Life Sci Alliance. 2020 Jan;3(1).

14. Cidre-Aranaz F, Ohmura S. Tumor Growth Analysis of Ewing Sarcoma Cell Lines Using Subcutaneous Xenografts in Mice. Methods Mol Biol Clifton NJ. 2021;2226:191–9.

15. Baldauf MC, Orth MF, Dallmayer M, Marchetto A, Gerke JS, Rubio RA, et al. Robust diagnosis of Ewing sarcoma by immunohistochemical detection of super-enhancer-driven EWSR1-ETS targets. Oncotarget. 2018 Jan 5;9(2):1587–601.

16. Orth MF, Hölting TLB, Dallmayer M, Wehweck FS, Paul T, Musa J, et al. High Specificity of BCL11B and GLG1 for EWSR1-FLI1 and EWSR1-ERG Positive Ewing Sarcoma. Cancers. 2020 Mar 10;12(3).

17. Volchenboum SL, Andrade J, Huang L, Barkauskas DA, Krailo M, Womer RB, et al. Gene Expression Profiling of Ewing Sarcoma Tumors Reveals the Prognostic Importance of Tumor-Stromal Interactions: A Report from the Children’s Oncology Group. J Pathol Clin Res. 2015 Apr;1(2):83–94.

18. Scotlandi K, Remondini D, Castellani G, Manara MC, Nardi F, Cantiani L, et al. Overcoming resistance to conventional drugs in Ewing sarcoma and identification of molecular predictors of outcome. J Clin Oncol Off J Am Soc Clin Oncol. 2009 May 1;27(13):2209–16.

19. Savola S, Klami A, Myllykangas S, Manara C, Scotlandi K, Picci P, et al. High Expression of Complement Component 5 (C5) at Tumor Site Associates with Superior Survival in Ewing’s Sarcoma Family of Tumour Patients. ISRN Oncol. 2011;2011:168712.

20. Postel-Vinay S, Véron AS, Tirode F, Pierron G, Reynaud S, Kovar H, et al. Common variants near TARDBP and EGR2 are associated with susceptibility to Ewing sarcoma. Nat Genet. 2012 Feb 12;44(3):323–7.

21. Stein CK, Qu P, Epstein J, Buros A, Rosenthal A, Crowley J, et al. Removing batch effects from purified plasma cell gene expression microarrays with modified ComBat. BMC Bioinformatics. 2015 Feb 25;16:63.

22. Irizarry RA. Exploration, normalization, and summaries of high density oligonucleotide array probe level data. Biostatistics. 2003 Apr 1;4(2):249–64.

23. Dai M, Wang P, Boyd AD, Kostov G, Athey B, Jones EG, et al. Evolving gene/transcript definitions significantly alter the interpretation of GeneChip data. Nucleic Acids Res. 2005 Nov 10;33(20):e175.

24. Stichel D, Schrimpf D, Casalini B, Meyer J, Wefers AK, Sievers P, et al. Routine RNA sequencing of formalin-fixed paraffin-embedded specimens in neuropathology diagnostics identifies diagnostically and therapeutically relevant gene fusions. Acta Neuropathol (Berl). 2019 Nov;138(5):827–35.

25. Orth MF, Surdez D, Marchetto A, Grossetête S, Gerke JS, Zaidi S, et al. Systematic multi-omics cell line profiling uncovers principles of Ewing sarcoma fusion oncogene-mediated gene regulation [Internet]. 2021 Jun [cited 2021 Aug 16] p. 2021.06.08.447518. Available from: https://www.biorxiv.org/content/10.1101/2021.06.08.447518v1
