# Supplementary material for: Integrative gene network and functional analyses identify a prognostically relevant key regulator of metastasis in Ewing sarcoma
Source: Mol Cancer. 2022 Jan 3;21:1. doi: 10.1186/s12943-021-01470-z (PMC8722160; doi:10.1186/s12943-021-01470-z)
Supplement: Supplementary file 2 — Additional file 2: [file 12943_2021_1470_MOESM2_ESM.docx]

**Supplementary Fig. 1 | TCF7L1 regulation in EwS.**

**a)** *TCF7L1* expression in 5 EwS wild type cell lines. *n*=*3* biologically independent experiments. **b)** *TCF7L1* and *EWSR1-ETS* expression in 5 EwS cell lines with conditional knock-down (KD) of EWSR1-ETS for 72h measured by qRT-PCR. *n*≥*3* biologically independent experiments. **c)** Left: *EWSR1-FLI1* and *TCF7L1* expression (Affymetrix microarrays) in A673/TR/shEF1 xenografts after 96 h of DOX treatment. Horizontal bars represent means, *n*=3 xenografts per group. *T*wo-sided independent *t*-test. Right: representative IHC for (EWSR1-)FLI1 and TCF7L1. Scale bar=20 µm. **d)** Profile of the *TCF7L1* locus in indicated EwS cells from published ChIP-seq data for *EWSR1-FLI1* and H3K4me3. Promoter region of *TCF7L1* is highlighted in red. **e)** Analysis of gain/no gain of chromosome 2 in a cohort of 115 EwS patients with paired *TCF7L1* expression (RNAseq). **f)** Pearson correlation of mean CNV scores at the *TCF7L1* locus and *TCF7L1* expression. **g)** Pearson correlation of mean methylation scores at CpG island in the *TCF7L1* promoter (chr2:85359957-85362593) and *TCF7L1* expression. **h)** Size-proportional Venn diagrams of genes concordantly regulated 96h after knockdown of *EWSR1-ETS* or upregulation of *TCF7L1* in TC-71 and SK-N-MC EwS cells. Minimum log_2_ fold-change ± 0.5. Fischer’s exact test.

**Supplementary Fig. 2 | TCF7L1 re-expression inhibits tumorigenesis *in vitro* and *in vivo.***

**a)** Viable cell count of TC-71 and SK-N-MC cells containing a DOX-inducible re-expression construct for an empty control 72h after treatment with or without DOX. Data are mean and SEM, *n*≥*3* biologically independent experiments. Two-sided Mann-Whitney test. **b)** Relative colony number of colony-forming assays (CFAs) of TC-71 and SK-N-MC cells containing a DOX-inducible re-expression construct for *TCF7L1*. Cells were grown either with or without DOX. *n*=4 biologically independent experiments. Two-sided Mann–Whitney test. **c)** Relative colony number of CFAs of TC-71 and SK-N-MC cells containing a DOX-inducible re-expression construct for an empty control. Cells were grown either with or without DOX. *n*=4 biologically independent experiments. Two-sided Mann-Whitney test. **d)** Relative percentage of area covered by colonies grown in soft-agar of TC-71 and SK-N-MC cells containing a DOX-inducible re-expression construct for an empty control. Cells were grown either with or without DOX. *n*=3 biologically independent experiments. Two-sided Mann-Whitney test. **e)** Representative images of *TCF7L1* expression on protein level by IHC on xenografted tissues from *in vivo* subcutaneous xenografts of TC-71 and SK-N-MC EwS cells with conditional re-expression of TCF7L1. **f)** Immuno Reactive Scores (IRS) of TCF7L1 expression in all subcutaneous xenografts of TC-71 and SK-N-MC EwS cells with conditional re-expression of TCF7L1 (*n*=7 animals/group). Horizontal bars represent mean. **g)** Subcutaneous growth of EwS xenografts of TC-71 and SK-N-MC cells containing a DOX-inducible re-expression construct for an empty control (arrow indicates start of DOX-treatment). Data are represented as means (*n*=5 animals/group). Two-sided Mann-Whitney test. **h)** *Ex vivo* analysis of mitotic index. Data are mean and SEM, *n*≥4 animals/group. **i)** Relative necrotic area of xenografted empty control TC-71 and SK-N-MC cell lines. Data are mean and SEM, *n*≥4 animals/group.

**Supplementary Fig. 3 | High TCF7L1 expression inhibits metastasis in EwS**

**a)** Kaplan-Meier plots showing event-free survival time of NSG mice bearing orthotopic xenografts of SK-N-MC cells containing a DOX-inducible re-expression construct for TCF7L1. After intra-osseous injection, mice were randomized and treated with either vehicle (–) or DOX (+). Event: first signs of limping. Log-rank (Mantel-Cox) test. **b)** Representative micrographs of HE from tibias obtained from both groups of mice orthotopically injected with SK-N-MC cells containing a DOX-inducible re-expression construct for TCF7L1. Mice were treated with either vehicle (–) or DOX (+). Scale bar=500 µm. EwS infiltrating area is shown in blue dots, black dots show bone tissue, green lines show muscle tissue and black lines show cartilage tissue. **c)** Graph depicts total number of macro-metastases counted after inner organ extraction in mice bearing orthotopically implanted TC-71 cells containing a DOX-inducible re-expression construct for TCF7L1. n=8 evaluated livers from independent animals in each group. **d)** Representative images from metastatic organs of mice bearing orthotopically implanted TC-71 cells containing a DOX-inducible re-expression construct for TCF7L1 in DOX (–) and DOX (+) conditions. **e)** Representative histological images of HE stainings from the evaluated organs. Scale bar=50 µm. M, metastasis; N, normal. **f)** mRNA expression of downregulated driver target genes of *TCF7L1* SK-N-MC and TC-71 EwS cell lines with either re-expression of wild type *TCF7L1* or its DNA-binding domain mutant (HMG-mut). *n*=*3* biologically independent experiments, unpaired two-sided t-test.
